# What to Say When Seeking Support Online: A Comparison Among Different Levels of Self-Disclosure

**DOI:** 10.3389/fpsyg.2020.00978

**Published:** 2020-06-03

**Authors:** Wenjing Pan, Bo Feng, V. Skye Wingate, Siyue Li

**Affiliations:** ^1^School of Journalism and Communication, Renmin University of China, Beijing, China; ^2^Department of Communication, University of California, Davis, Davis, CA, United States; ^3^College of Media and International Culture, Zhejiang University, Hangzhou, China

**Keywords:** depth of self-disclosure, perceived anonymity of the other, support-seeking, support-provision, person-centeredness, politeness

## Abstract

The current study examined the effect of exposure to online support-seeking posts containing different levels of depth self-disclosure (baseline, peripheral, core) affecting the quality (person-centeredness and politeness) of participants’ support-provision messages. Participants of the study were assigned to the role of a support-provider. Compared to participants who read support-seeking posts with baseline and core self-disclosure, participants who read support-seeking posts with peripheral self-disclosure rated the support-seekers as less anonymous. Compared to participants who read support-seeking posts in the baseline condition, participants who read the support-seeking posts with peripheral self-disclosure wrote support-provision messages with higher level of person-centeredness and politeness. Participants’ perceived anonymity of the support-seekers mediated the effect of the depth of self-disclosure on the politeness of the response messages.

## Introduction

Supportive interactions are ubiquitous in people’s everyday lives, and the positive impacts of social support on people’s mental and physical well-being have been well documented in the literature (for a review, see MacGeorge et al., 2011). With the advancement in Internet technology, people are seeking social support more often in online support groups, communities, and forums due to the anonymity, asynchronicity, large audience, and ease of access of online venues (Wright, 2016). Online support groups provide individuals with opportunities to learn from others who share similar experiences and help reduce the stress caused by negative life events (Tanis, 2008).

Earlier research on online support groups has investigated several important aspects about online support, including (a) the association between participation in online support groups and the subsequent health outcomes (e.g., Mo and Coulson, 2013; Batenburg and Das, 2015), (b) typologies of solicited and provided social support in online support groups (see Rains et al., 2015 for a review), (c) types of discussion topics (Eichhorn, 2008; Coursaris and Liu, 2009), and (d) factors promoting/inhibiting participation in online support groups (Chung, 2013; Wright and Rains, 2013). More recently, some scholars began to pay research attention to the link between support-seeking strategies and support-provision outcomes in online support groups (e.g., Li and Feng, 2015; Feng et al., 2016; Pan et al., 2018).

Given the fact that there are numerous people posting support-seeking requests online at any given moment every day, one research question with theoretical and practical importance is to investigate what motivates people to take the time and energy to respond to and help a strange, distant person’s support-seeking requests in cyberspace (Feng et al., 2016). Internet technology enables users to communicate with others anonymously. The anonymity of online venues makes it easier for people who do not feel comfortable discussing private issues with others in face-to-face (FtF) situations to open to others and seek support (Wright, 2002). Online anonymous communication is especially beneficial to support-seekers who have health concerns and related stigma toward illness and disease. However, from the message receiver’s perspective, support-seeking messages from an anonymous user may not receive adequate attention due to a lack of perceived identifiability. The current study takes particular interest in the following question: “How would online support-seekers’ depth of self-disclosure influence their chances of obtaining high-quality supportive messages from others?” In the following section, we first offer a distinction between the perceived anonymity of the *self* and the *other*. Then, we present the theoretical frameworks that guide our predictions of how a support-seeker’s self-disclosure depth may influence the perceived anonymity of the other, as well as the quality of received support in terms of the degree of person-centeredness and politeness. Lastly, we present an empirical study to test the predictions.

### Perceived Anonymity in Text-Based Computer-Mediated Communication

As noted earlier, one of the key contributors to the popularity of online support groups is anonymity, which can be defined as the state where a person is not identifiable (Marx, 1999). Although prior to the advent of the Internet, individuals could communicate anonymously through telephone or letter, the advancement in Internet technology and the popularity of text-based online communication provides people with easy access to anonymous communication. In text-based online forums, anonymity has been identified as an important factor in facilitating self-disclosure (Qian and Scott, 2007; Rains, 2014). Past theorizing and research have also examined the influence of anonymity on other types of behaviors such as computer-mediated group communication (e.g., Rains, 2007), online privacy, and trust management (e.g., Joinson et al., 2010). In the current study, we take a close look at the concept of anonymity and its implications for online supportive communication from the perspective of the fundamental process of social perceptions. Social perceptions, including perceptions individuals have regarding themselves, others, social relationships, and social institutions, can impact various interpersonal communication outcomes (Burleson, 2010). As is well known, an individual can form perceptions of him/herself from the perspective of a message sender or a message receiver. The same individual can also form perceptions of others when others are acting as message senders or receivers. Seen in this light, previous theorizing regarding perceived anonymity has focused almost exclusively on the perceived anonymity of *self* as message sender. In the current study, we focus on perceived anonymity of the *other* as message sender.

To date, most theoretical discussions of the anonymity feature of online support groups have focused on the message sender’s perspective, highlighting the idea that the anonymous feature of online support groups facilitates honest self-disclosure among users and provides them with a sense of security (Wright and Bell, 2003; Suler, 2004; Li et al., 2015). Correspondingly, scholars have focused on the effects of the message sender’s perceptions of their own anonymity on how they communicate with others in text-based computer-mediated communication (CMC) situations (e.g., Qian and Scott, 2007; Tanis and Postmes, 2007; Hollenbaugh and Everett, 2013). From the message sender’s perspective, users prefer to stay anonymous because it helps to protect their privacy and offer them a sense of security. The online disinhibition effect posits that “When people have the opportunity to separate their actions online from their in-person lifestyle and identity, they feel less vulnerable about self-disclosing and acting out” (Suler, 2004, p. 322). This type of anonymity is found to be strategically used and fostered self-disclosure among individuals with illness-related stigma (Rains, 2014).

With a few exceptions (Rains, 2007; Rains and Scott, 2007), little empirical research has examined the effect of perceived anonymity of the *other as message sender.* Available research shows that anonymity can function differently depending on the origin of anonymity. As noted by Rains and Scott (2007), although anonymity enables a message sender to comfortably discuss sensitive topics, the receivers may question the credibility of the sender and, in turn, produce messages with low quality. In interpersonal communication, one’s perceived anonymity of the source can play an important role in his/her evaluation of the credibility or trustworthiness of the source (Rains, 2007). From the perspective of Uncertainty Reduction Theory (Berger, 1987), messages produced by an anonymous source can increase uncertainty compared with messages from an identifiable source. The fundamental assumption of Uncertainty Reduction Theory is that uncertainty is unpleasant and individuals seek to reduce it (Berger, 1987). When the source is identifiable, individuals tend to perceive the source as more trustworthy and familiar. The reduced uncertainty and increased trust can elicit positive impressions and facilitate more engaged interactions (Derlega et al., 1993).

### Perceived Anonymity and Self-Disclosure of Other

The concept of anonymity is closely connected with the notion of identity cues. Some types of identity cues can be utilized to pinpoint a person (e.g., legal name and residential address) while other identity cues may not be useful to know a person’s identity (e.g., social categorization) (Qian and Scott, 2007). In online text-based CMC, identity cues are often revealed through self-disclosure. In general, self-disclosure refers to the revelation of personal information (Derlega et al., 1993). Self-disclosure can differ along two dimensions: breadth and depth. Breadth of self-disclosure refers to the various topics disclosed, while depth of self-disclosure refers to the intimacy of the information disclosed (Altman and Taylor, 1973). There are three layers within the depth dimension of self-disclosure, namely, peripheral layer (e.g., biographical data such as name, age, and gender), intermediate layer (e.g., attitudes, values, and opinions), and core layer (e.g., beliefs, needs, and fears) (Altman and Taylor, 1973). Peripheral self-disclosure containing personal identity cues such as one’s name, location, and occupation should reduce one’s perceived anonymity of the other as message sender. Core self-disclosure containing information about one’s self-concept may be more private and intimate; however, it cannot be adopted to identify the unique source of information. Therefore, individuals who engage in peripheral self-disclosure containing personal identity cues should be viewed as less anonymous compared to individuals who engage in core self-disclosure. In the current study, since a support-seeking post will necessarily contain descriptions of the problematic situation the support-seeker is experiencing, it will serve as a “baseline self-disclosure” condition as the control condition. Thus, the following hypothesis was proposed:

H1: Support-providers will perceive lower level of anonymity of support-seekers who engage in peripheral self-disclosure than support-seekers who engage in (a) core self-disclosure or (b) baseline self-disclosure.

### Quality of Support-Provision Messages: Verbal-Person-Centeredness and Politeness

Support-provision messages can be defined as communicative behaviors enacted by one party with the intention of benefiting or helping another (MacGeorge et al., 2011). Support-provision messages vary in terms of forms, lengths, and, most importantly, quality. For example, some forms of support messages are more effective than others in comforting distressed individuals and helping them to solve problems. The quality of support messages has been defined in terms of person-centeredness, which refers to the extent to which a support message explicitly acknowledges the target’s feelings and helps the target reappraise the distressing situation (MacGeorge et al., 2011). Recent research has identified the exhibition of politeness as another dimension of support-provision messages distinct from person-centeredness (Li and Feng, 2015; Feng et al., 2016). Attending to support-seekers’ face needs can help alleviate their stress and facilitate their problem solving (MacGeorge et al., 2004). Therefore, the current study examines the quality of support-provisions messages in terms of both person-centeredness and politeness.

Person-centeredness is a general index of the quality of messages and highly person-centered messages adapt to the specific contexts of communication, including the primary communicative goal being pursued (e.g., comforting, persuading, informing). Support messages with high person-centeredness acknowledge and legitimize other’s feelings while support messages with low person-centeredness criticize and challenge the legitimacy of the distressed other’s feelings (MacGeorge et al., 2011). Previous studies have established that support messages with high person-centeredness can reduce emotional distress and facilitate coping (High and Dillard, 2012). Support messages with high person-centeredness also have long-term positive effects in terms of support receivers’ impressions of the support quality and improvement in terms of coping with stressful situations (High and Solomon, 2014).

Politeness is a universal phenomenon and it is a function of individuals’ need for their face (Brown and Levinson, 1987). According to Politeness Theory (Brown and Levinson, 1987), there are two types of face, positive face (one’s desire to have his/her image and behavior to be recognized by others) and negative face (one’s desire to assert autonomy and rights). Politeness reflects other’s recognition of the positive image of the target (positive politeness) and attempts to minimize the imposition of autonomy and freedom of choice on the target (negative politeness) (Brown and Levinson, 1987). Support messages addressing receiver’s positive and negative face needs are considered as more sensitive, appropriate, and effective (Feng et al., 2016). Support messages that fail to address or even threaten the receiver’s face needs tend to be evaluated as not helpful (MacGeorge et al., 2011).

Previous research on online support-seeking and provision finds that support-providers’ certain perceptions regarding the support-seeker (e.g., social presence, trust) may affect their motivation to provide support-provision messages with high quality (Feng et al., 2016; Pan et al., 2018). In this study, we investigate whether the depth of self-disclosure in support-seeking posts would affect the quality of the response messages through the support-providers’ perceived anonymity of the support-seeker. The cues-filtered-out perspective can serve as a useful framework.

The term “cues-filtered-out” perspective was coined by Culnan and Markus (1987) to refer to a group of theories with the basic assumption that text-based CMC lacks non-verbal cues and the related social functions involving those cues will be impaired. Based on the Social Presence Theory (Short et al., 1976) and the Cuelessness Model (Rutter and Stephenson, 1979), the quality of interpersonal communication is impaired when identity cues are absent. Identity cues, or “social context cues,” usually include information regarding the demographic (e.g., gender, age, race, location) and personal characteristics (e.g., accent, tone, appearance) of interaction partners (see Walther and Parks, 2002, for a review). Compared to FtF interaction, text-based CMC is characterized by the lack of social context cues. In asynchronous text-based online venues, social presence is further diminished by the lack of direct, two-way interaction (Taylor, 2011). The lack of social context cues and personal identity cues can lead to negative outcomes such as poor information quality, deception, and even flaming (Douglas and McGarty, 2002).

In anonymous online communication venues, the presence of personal identity cues can promote participation in online discussion (Donath, 1998) and elicit enhanced awareness and positive perceptions of others (Tanis and Postmes, 2007). For example, one study found that support-seeking posts with more personal identity cues (first name ID and portrait picture) yielded higher quality of support-provision messages compared to those with fewer personal identity cues (no name ID and no portrait picture).

Peripheral self-disclosure including demographic information (e.g., name, location, and occupation) contains more unique personal identity cues compared to core self-disclosure including self-concept information (e.g., fear and value). In FtF settings, disclosing one’s self-concept information can make the person more vulnerable to others compared to disclosing peripheral information (Mesch and Beker, 2010). In the context of online forums where anonymity is the norm, support-seekers engaging in peripheral self-disclosure should be viewed as less anonymous compared to support-seekers engaging in core self-disclosure. Peripheral self-disclosure containing demographic and biographical information in online anonymous settings can expose the person’s identity to others, thus making the person more vulnerable. Peripheral self-disclosure containing identity cues can yield enhanced awareness and more positive perceptions of the discloser compared to situations where identity cues were scarce or absent (Tanis and Postmes, 2007). Disclosing personal identity cues can reduce message recipient’s uncertainty regarding the message source and build trust among communicators (Derlega et al., 1993; Marx, 2004; Rains and Scott, 2007). From the perspective of Uncertainty Reduction Theory (Berger, 1987), the anonymity of the message sender produces more uncertainty in terms of the source’s identity. When the message sender is anonymous, message receivers will experience more uncertainty regarding the source’s identity and therefore less motivation to engage in warm, intimate forms of communication (Rains, 2007). Potential support-providers should be more motivated to produce support-provision messages with better quality when the support-seekers are less anonymous. In turn, the decreased uncertainty and perceived anonymity of the support-seekers will promote potential support-providers to form positive impressions of the support-seekers and provide pro-social responses such as writing response messages with better quality. Therefore, we propose the following hypotheses:

H2: Support-providers’ responses to support-seeking posts containing peripheral self-disclosure will show higher level of person-centeredness than responses to support-seeking posts containing (a) core self-disclosure and (b) baseline self-disclosure.
H3: Support-providers’ responses to support-seeking posts containing peripheral self-disclosure will show higher level of politeness than responses to support-seeking posts containing (a) core self-disclosure and (b) baseline self-disclosure.
H4: Support-providers’ perceived anonymity of the support-seekers will mediate the effect of depth of self-disclosure on the level of (a) person-centeredness and (b) politeness of response messages.

## Materials and Methods

### Participants

A total number of 703 undergraduate students from a comprehensive university located in the west coast of the United States participated in this study. In exchange, they were offered extra credits for participation. The mean age of the participants was 20.44 (SD = 2.24). 69.3% of the participants (*n* = 487) were females and 30.7% (*n* = 216) were males. In terms of participants’ year in school, 23.2% (*n* = 163) were freshman, 21.6% (*n* = 152) were sophomore, 28.6 (*n* = 201) were junior, and 26.6% (*n* = 187) were senior.

### Experimental Design

This study employed a 3 (depth of self-disclosure: peripheral vs. core vs. baseline) × 2 (problem type: job vs. major) factorial design. The baseline condition containing a simple description of the problematic situation the support-seeker experienced was included as a control condition to increase the ecological validity of the study design. The mere description of the problem could be viewed as self-disclosure with moderate depth. The peripheral self-disclosure condition included the support-seeker’s demographic information including geographic location, major, and occupation plus the description of the problem. The core self-disclosure condition included disclosure of the support-seeker’s self-concept including values, beliefs, and fears plus the description of the problem. The length of the messages was kept equal within each level of the depth of self-disclosure condition. The six messages were included in Supplementary Appendix A.

All six messages were pre-tested to check if the manipulation of the depth of self-disclosure was perceived as intended. A separate sample of 113 students evaluated the perceived privacy of the messages. Two items on an 11-point scale (“How private do you think the message is” and “How likely would you share information similar to what is disclosed in the message”) were used to measure the perceived privacy of the messages they were presented to.

The support-seeking posts containing core self-disclosure (*M* = 5.25, SD = 3.36) were perceived as more private compared to support-seeking posts containing baseline self-disclosure (*M* = 4.66, SD = 2.96), *t*(112) = 3.16, *p* < 0.001, and peripheral self-disclosure (*M* = 4.04, SD = 2.93), *t*(112) = 5.59, *p* < 0.001.

### Procedure

Upon arrival at the lab, each participant was presented with a consent form. They were told that their participation was voluntary and they can leave the experiment at any time during the study. Participants were individually assigned to a cubicle with a computer and randomly assigned to an experimental condition. An interactive online forum was used in the experiment to maximally resemble a real-life experience of participating in online forum discussion. All participants were assigned to the role of a support-provider. Participants were first directed to read the online forum support-seeking post and write a response message afterward. There was no minimal or maximal length of participants’ responses and there was no time limit for them to reply. Once they clicked “post a reply,” their own responses would appear underneath the support-seeking post. After that, participants were directed to an online survey on an online survey site “Qualtrics” to complete a questionnaire. Once they complete the questionnaire, they were thanked and debriefed by the research assistants.

### Measures

#### Perceived Anonymity of the Support-Seeker

Five items on a nine-point Likert scale were adapted (Marx, 1999) to measure participants’ perceived anonymity of the support-seeker. Participants were asked to indicate the degree (1 = *completely disagree*, 9 = *completely agree*) to which they felt they could identify the following characteristics about the poster (see Supplementary Appendix B for the detailed scale). The five items showed good internal consistency (*M* = 5.09, SD = 1.21, α = 0.72).

#### Person-Centeredness of Response Messages

The level of person-centeredness of participants’ response messages was coded based on the nine-level hierarchy developed by Applegate (1980) and Burleson (1982). Messages that deny the target’s feelings by condemning them, challenging their legitimacy, or ignoring them were coded in one of the three levels within Major Level 1 (levels 1–3) (Burleson, 1982). Messages that implicitly recognize the target’s feelings by attempting to distract the target, offer expressions of sympathy, or present explanations of the situation were coded in one of the three levels within Major Level 2 (levels 4–6) (Burleson, 1982). Messages that explicitly recognize and legitimize the target’s feelings by helping the target to articulate them, elaborating reasons why the feelings might be felt, or assisting the target to see how the feelings fit in a broader context were coded in one of the three levels within Major Level 3 (levels 7–9) (Burleson, 1982). Coding manual of person-centeredness and sample messages can be found in Supplementary Appendix C.

#### Politeness of Response Messages

For politeness, each message was coded based on the scheme developed by Feng et al. (2016). Twelve positive and seven negative politeness strategies were included in the coding scheme. Overall politeness was calculated as the sum of positive and negative politeness scores. Coding manual of politeness and examples can be found in Supplementary Appendix D.

#### Intercoder Reliability

Three student researchers coded a random sample of about 30% of the data independently. For person-centeredness, the intercoder reliability (intraclass correlation coefficient) was 0.80, and for politeness, the intercoder reliability was 0.84. Disagreements were resolved through discussions. The remaining coding was split evenly among the three assistants.

## Results

Preliminary analyses were conducted to examine if the type of problem disclosed in the support-seeking posts had any effect on participants’ perceived anonymity of the support-seeker and the level of person-centeredness and politeness of the response messages. Problem type of the support-seeking posts did not affect the perceived anonymity of the support-seeker, *F*(1,700) = 1.01, *p* = 0.32, *ns*, the level of person-centeredness of the response messages, *F*(1,700) = 0.15, *p* = 0.69, *ns*, or the politeness of the response messages, *F*(1,700) = 0.39, *p* = 0.53, *ns*. Furthermore, problem type did not moderate the effect of depth of self-disclosure on perceived anonymity, *F*(1,700) = 0.51, *p* = 0.60, *ns*, on the person-centeredness of the response messages, *F*(1,700) = 1.30, *p* = 0.27, *ns*, or on the politeness of the response messages, *F*(1,700) = 1.36, *p* = 0.26, *ns*. Therefore, problem type of the support-seeking posts was not included in the subsequent analyses. Descriptive statistics of key variables were included in Table 1, and the results of the statistical tests were included in Table 2.

**TABLE 1:** Means and standard deviations of the key variables.

Manipulation of depth of self-disclosure	Baseline self-disclosure		Peripheral self-disclosure		Core self-disclosure	
			
	Mean	SD	Mean	SD	Mean	SD
Perceived anonymity	5.01	1.21	4.63	1.20	5.11	1.12
Person-centeredness	4.38	1.28	4.76	1.38	4.84	1.26
Politeness	3.55	2.36	4.22	2.48	3.88	2.43

**TABLE 2:** Effects of depth of self-disclosure on key outcome variables.

Independent variable	Dependent variable	*F* value	Significance (*p*-value)	Effect size ($\eta_{p}^{2}$)
Depth of self-disclosure	Perceived anonymity	10.88	<0.001	0.03
	Person-centeredness	4.84	<0.01	0.01
	Politeness	5.72	0.003	0.02

H1(a) and H1(b) were concerned with the effect of depth of self-disclosure on the perceived anonymity of the support-seekers. These hypotheses were tested through ANOVA with the depth of self-disclosure as the independent variable and perceived anonymity of the support-seekers as the dependent variable. A significant main effect was observed for the depth of self-disclosure on the participants’ perceived anonymity of the support-seekers, *F*(2,700) = 10.88, *p* < 0.001, $\eta_{p}^{2}=0.03$. A Tukey LSD *post hoc* test showed that participants who read support-seeking posts containing peripheral self-disclosure (*M* = 4.63, SD = 1.20) indicated lower perceived anonymity of the support-seekers compared to those who read support-seeking posts containing core self-disclosure (*M* = 5.11, SD = 1.12), *p* < 0.001, and those who read support-seeking posts containing baseline self-disclosure, (*M* = 5.01, SD = 1.21), *p* < 0.001. H1(a) and H1(b) were supported.

H2(a) and H2(b) were concerned with the effect of depth of self-disclosure on the person-centeredness of participants’ support-provision messages. These hypotheses were tested through ANOVA with the depth of self-disclosure as the independent variable and level of person-centeredness of the response messages as the dependent variable. First, depth of self-disclosure had a significant main effect on the level of person-centeredness in the response messages, *F*(2,700) = 4.84, *p* < 0.01, $\eta_{p}^{2}=0.01$. A Tukey LSD *post hoc* test showed that participants who read support-seeking posts containing peripheral self-disclosure (*M* = 4.76, SD = 1.38) wrote response messages with higher level of person-centeredness compared to the ones who read support-seeking posts containing baseline self-disclosure (*M* = 4.38, SD = 1.28), *p* < 0.01. Compared to participants who read support-seeking posts containing baseline self-disclosure (*M* = 4.38, SD = 1.28), those who read support-seeking posts containing core self-disclosure (*M* = 4.84, SD = 1.26) wrote response message with higher level of person-centeredness, *p* < 0.001. Participants who read support-seeking posts containing peripheral self-disclosure (*M* = 4.76, SD = 1.38) did not differ from the ones who read support-seeking posts containing core self-disclosure (*M* = 4.84, SD = 1.26) in terms of the person-centeredness of their response messages. H2(a) was not supported while H2(b) was supported.

H3(a) and H3(b) were concerned with the effect of depth of self-disclosure on the politeness of participants’ response messages. These hypotheses were tested through ANOVA with the depth of self-disclosure as the independent variable and level of politeness of the response messages as the dependent variable. Depth of self-disclosure on the support-seeking posts had a significant main effect on the level of politeness in the response messages, *F*(2,700) = 5.72, *p* = 0.003, $\eta_{p}^{2}=0.02$. A Tukey LSD *post hoc* test revealed that participants who read support-seeking posts containing peripheral self-disclosure (*M* = 4.22, SD = 2.48) wrote response messages with higher politeness compared to the ones who read support-seeking posts containing baseline self-disclosure (*M* = 3.56, SD = 2.36), *p* < 0.01. Participants who read support-seeking posts containing baseline self-disclosure (*M* = 3.56, SD = 2.36) did not differ from the ones who read support-seeking posts containing core self-disclosure (*M* = 3.88, SD = 2.43) in terms of the politeness of their response messages, *p* = 0.17. Participants who read support-seeking posts containing peripheral self-disclosure (*M* = 4.22, SD = 2.48) did not differ from the ones who read support-seeking posts containing core self-disclosure (*M* = 3.88, SD = 2.43) in terms of the politeness of their response messages. H3(a) was not supported while H3(b) was supported.

H4 was regarding the mediating role of perceived anonymity of the support-seekers on the effect of depth of self-disclosure in affecting (a) person-centeredness and (b) politeness of the response messages. Six mediation analyses were carried out using PROCESS—a statistical analysis program adopting bootstrapping to detect indirect effect (Hayes, 2013). The first three mediation analyses focused on the mediation effect of perceived anonymity of the support-seekers on the link between depth of self-disclosure and person-centeredness of response messages. The indirect effect of depth of self-disclosure (peripheral vs. core) on the person-centeredness of participants’ response messages was not significant [*b* = 0.00, SE = 0.03, 95% CI = (−0.565, 0.479)] after bootstrapping with 5000 resamples. The indirect effect of depth of self-disclosure (peripheral vs. baseline) on the person-centeredness of participants’ response messages was not significant [*b* = 0.01, SE = 0.01, 95% CI = (−0.0132, 0.269)]. The indirect effect of depth of self-disclosure (core vs. baseline) on the person-centeredness of participants’ response messages was not significant [*b* = −0.01, SE = 0.01, 95% CI = (−0.0403, 0.0055)]. H4(a) was not supported.

The other three mediation analyses were regarding the mediation effect of perceived anonymity of the support-seekers on the link between depth of self-disclosure and the politeness of response messages. The indirect effect of depth of self-disclosure (peripheral vs. core) on the politeness of participants’ response messages was significant [*b* = 0.05, SE = 0.04, 95% CI = (0.0037, 0.1459)]. The indirect effect of depth of self-disclosure (peripheral vs. baseline) on the politeness of participants’ response messages was significant [*b* = 0.04, SE = 0.02, 95% CI = (0.0081, 0.0896)]. The indirect effect of depth of self-disclosure (core vs. baseline) on the politeness of participants’ response messages was not significant [*b* = 0.01, SE = 0.01, 95% CI = (−0.0369, 0.0138)]. The results indicated that, in comparison to core and baseline self-disclosure, the effect of peripheral self-disclosure on the politeness of response messages was mediated by the perceived anonymity of the support-seekers. H4(b) was supported.

## Discussion

As online support forums are becoming more and more commonly used, it is crucial to understand how forum users can benefit more from taking part in these venues of supportive communication. However, the benefits of turning to cyberspace for help are far from guaranteed. Many requests for support are not met or simply ignored. For example, recent research on an online depression forum found that only less than 10% of the posts have received at least one reply from others (Pan et al., 2018). Given the fact that numerous new postings are shared online daily and the limited time, interest, and motivation individuals have for attending to unknown others’ needs in the cyberspace, it is especially crucial for an online support-seeker to be aware of possible strategies that can be utilized to increase their chance of receiving the attention and help they need from others, as well as receiving good quality support. This study focused on the effect of a distinctive feature of support-seeking messages, namely, depth of self-disclosure, on the quality of the support-provision messages. The proposed effect was further examined through the mediating role of perceived anonymity of the support-seeker.

Our results showed that participants with the roles of support-providers who read the support-seeking posts with peripheral self-disclosure perceived the support-seekers as less anonymous compared to the ones containing baseline and core self-disclosure. Support-seeking posts containing demographic information regarding one’s location and occupation elicited lower perceived anonymity of the support-seekers. As argued by Qian and Scott (2007), these types of identity information can be used to identify the source more accurately than other types of information. This is also in line with Marx’s (2004) 11-type topology regarding one’s identity information. Individual identification (the “who” question) and geographical/locational identification (the “where” and “how to reach” question) were considered as important information to identify someone (Marx, 2004, p. 144). Rains and Scott (2007) noted that information regarding one’s individual identification (name) and location are two of the most important pieces of identity information affecting the perceived anonymity of the source. Furthermore, these two factors are related to discursive and physical anonymity and they have been central to research in group-based CMC (Rains and Scott, 2007).

While a plethora of studies have focused on the positive influences of anonymity such as reducing the stigma associated with illness, mitigating status differences, and reducing pressure coming from possible retribution of online anonymous communication (Postmes and Lea, 2000; Rains, 2014), our study showed that anonymity can also produce negative influences on one’s perceptions of the other. The Dual-Process Theory of Supportive Messages argues that individuals’ social perception capabilities and motivations both contribute to the production of person-centered messages (Burleson, 2009). Although individuals’ perceptual capabilities are relatively stable, motivation to produce person-centered messages tends to vary across situations. The Dual-Process Theory seeks to identify “cues” other than message content that may trigger heuristics in changing people’s cognitions, affects, and environment (Burleson, 2009). In text-based CMC, anonymity can serve as a cue impacting motivation to produce higher- or lower-quality support messages. One pragmatic implication of this finding is that it is important for online forum users to find a balance between users’ perceived anonymity of self as the sender and their perceived anonymity of other as message sender. On one hand, perceived anonymity of self can encourage honest self-disclosure. On the other hand, anonymous sources will also be perceived as less trustworthy and competent. For potential support-seekers, they could use the anonymity feature strategically when seeking support in online forums. Online support forums can take various forms and cover different topics. There are generic forums such as Quora and there are forums designed for specific topics such as weight-loss support forums or cancer support forums. The problems people discuss on those forums and the sensitivity of the problems also vary. For example, people may have greater concerns about their anonymity when discussing stigmatized health-related issues such as AIDs or depression. When the topic of discussion is not sensitive to the support-seekers, they can engage in more peripheral self-disclosure in order to receive better quality support-provision messages.

Consistent with prior research on text-based CMC, our results showed that personal identity cues in the form of peripheral self-disclosure in support-seeking posts elicited lower perceived anonymity, which, in turn, led potential support-providers to provide messages with higher level of politeness. The quality of support-provision messages is a multi-facet concept with various aspects and dimensions. For example, scholars taking the psychological perspective have focused on the perceived availability or the quantity of provided support. Scholars taking the communication perspective have focused on the content of support-provision messages, highlighting person-centeredness as the key indicator of the quality of support-provision messages (Bodie et al., 2012; Li and Feng, 2015; Feng et al., 2016). Similarly, the current study adopted the person-centeredness and politeness to measure the quality of response messages. In the current study, the perceived anonymity of the support-seeker only mediated the effect of peripheral self-disclosure on the politeness of the support-provision messages. This finding suggests that when potential support-providers can identify the identity of the support-seeker, they were promoted to write polite response messages demonstrating their concerns of support-seekers’ face. However, they were less concerned with providing response messages demonstrating high level of person-centeredness. One possible explanation is that the perceptions regarding the source can only affect certain aspects of message features such as politeness. Relatedly, the source characteristic may only affect the production of supportive messages to a certain extent.

One caveat regarding the phased nature of social penetration process should be noted. Although the current study focused on examination of “one shot” interaction between support-seeker and support-provider(s) and therefore does not allow us to investigate the phases through which online relationships develop, it did not ignore or deny Social Penetration Theory’s proposition that disclosure in relationship development is a phased act. Our speculation is that disclosure in anonymous online environments is more likely to begin with intermediate or core information (i.e., non-demographic information) and as an online relationship advances to a higher level, relational partners are more likely to engage in disclosure of demographic information such as full name, occupation, location, etc. In other words, we expect that the trajectory of disclosure in online relationship development will be different (if not entirely opposite) to that in FtF settings. Of course, this proposition remains speculative at this point and awaits future empirical examination.

This study has several limitations. First, only one type of anonymity, namely, perceived anonymity of the other as sender, was examined in the study. Participants were asked to read a support-seeking post in an online anonymous support forum and provide their response afterward. When providing the response messages, participants of the study were not asked to provide any identity information about themselves. However, the perceived anonymity of the participants themselves may influence their perceived anonymity of the other as message sender. Future study should also include measurement of participants’ perceived anonymity of themselves and see if the two types of perceived anonymity may interact with each other.

Second, in order to examine the quality of response messages, participants in this study were assigned the role of a support-provider and they were required to write response messages. However, in real-life situations, many forum users are lurkers and never contribute to forum discussions (Feng et al., 2016). Other than the quality of response messages, future studies can examine the likelihood of response as a potential support-provision outcome.

Third, this study was conducted in the lab. The forum used in this study was designed for the purpose of the study; as a result, participants had no prior experience using the forum or any interaction history with the support-seeker. In real-life situations, forum users’ experience or perceptions about others (such as support-seekers’ old posts, profile pictures) may also affect how they perceive each other as anonymous or not. A direction for future research is to reexamine the research questions using a more naturalistic online experimental design.

Fourth, our study adopted a 3 (self-disclosure: peripheral vs. core vs. baseline) × 2 (problem type: job vs. major) factorial design. In other words, we had a total of six different messages, although each participant was randomly assigned to read one of the six messages. We included two problem types to help assess the generalizability and replicability of our findings. Although our findings pertaining to the effect of self-concept disclosure were consistent across the two problem types, we are not in a position to exclude the possibility that future experiments using different manipulations or problem types will generate different patterns of findings.

The findings of the current study offer several practical suggestions for individuals who turn to online support groups for help. Our study suggests that the inclusion of certain types of personal identity cues can be employed as an effective online support-seeking strategy. It is worth noting, however, that some personal identity cues may render individuals more vulnerable than others and therefore people should exercise caution while making choice about the types of personal identity cues to disclose. For example, revealing one’s home address, full legal name, or date of birth will certainly bring much greater risk than revealing one’s state of residence or first name. To the extent that support-seekers include some relatively superficial identity information without jeopardizing their privacy, they are more likely to receive better quality response messages from others.

## Data Availability Statement

The datasets generated for this study are available on request to the corresponding author.

## Ethics Statement

The studies involving human participants were reviewed and approved by the Institutional Review Board (IRB) of UC Davis. The patients/participants provided their written informed consent to participate in this study.

## Author Contributions

WP and BF contributed conception and design of the study. WP organized the experiment, performed the statistical analysis, and wrote the first draft of the manuscript; VW trained coders and organized the coding process. SL designed and implemented the materials. All authors wrote sections of the manuscript, contributed to manuscript revision and, read and approved the submitted version.

## Conflict of Interest

The authors declare that the research was conducted in the absence of any commercial or financial relationships that could be construed as a potential conflict of interest.
