# Supplementary material for: What to Say When Seeking Support Online: A Comparison Among Different Levels of Self-Disclosure
Source: Front Psychol. 2020 Jun 3;11:978. doi: 10.3389/fpsyg.2020.00978 (PMC7283557; doi:10.3389/fpsyg.2020.00978)
Supplement: Supplementary file 1 [file Data_Sheet_1.docx]

Appendix A. Support-seeking Messages Containing Different Levels of Depth of Self-disclosure

| Problem type | Job | Major |
| --- | --- | --- |
| Baseline self-disclosure | So I’ve been working in retail at a phone store. The new manager was a sales consultant who was given the management position by the owner… I’m the top sales consultant exceeding all targets and yet I got much less than that person. When the management position came up we were told we would all get interviews. The interview never happened. I don’t know how to deal with the situation. | So I am a Philosophy major at college. If I continue, I will be able to graduate next year. However, I’ve realized that career-wise philosophy isn’t very promising. So I’ve been thinking of changing my major to environmental science and I’m also interested in that area. But if I change major, it will probably take me two extra years to graduate. I don’t know how to deal with the situation. |
| Peripheral self-disclosure | So I graduated from Michigan State with a bachelor’s degree in Business Management. Since then I’ve been working in retail at a phone store called Cricket Wireless in Madison, Wisconsin. The new manager was a sales consultant who was given the management by the owner…I’m the top sales consultant exceeding all targets and yet I got much less than that person. When the management position came up we were told we would all get interviews. The interview never happened. I don’t know how to deal with the situation. | So I come from Wisconsin Madison and I am now a Philosophy major at Michigan State University. If I continue, I will be able to graduate next year. However, I’ve realized that career-wise philosophy isn’t very promising. So I’ve been thinking of changing my major to environmental science and I’m also interested in that area. But if I change major, it will probably take me two extra years to graduate. On top of that, I am already older than most of my peers (turning 24 very soon). I don’t know how to deal with the situation. |
| Core  self-disclosure | So I’ve been working in retail at a phone store. I see myself as a hardworking person and I believe in the values of equity and integrity. The new manager was a sales consultant who was given the management position by the owner… I’m the top sales consultant exceeding all targets and yet I got much less than that person. When the management position came up we were told we would all get interviews. The interview never happened. I don’t know how to deal with the situation. I fear that if I confront the owner about this, I would be fired. | So I am a Philosophy major at college. I’ve wanted to become a philosopher since I was young. If I continue, I will be able to graduate next year. However, I’ve realized that career-wise philosophy isn’t very promising. So I’ve been thinking of changing my major to environmental science and I’m also interested in that area. But if I change major, it will probably take me two extra years to graduate. Although I see myself as a hard working person, I fear that I won’t do well in the new major. I don’t know how to deal with the situation. |

Appendix B. Scale for Perceived Anonymity

Please use the scale below to indicate the degree to which you felt you could identify the following characteristics about the poster.

1 = completely disagree; 9 = completely agree

| 1. A way to locate the poster | 1 | 2 | 3 | 4 | 5 | 6 | 7 | 8 | 9 |
| --- | --- | --- | --- | --- | --- | --- | --- | --- | --- |
| 2. Patterns in the poster’s behavior | 1 | 2 | 3 | 4 | 5 | 6 | 7 | 8 | 9 |
| 3. Information about the social categories to which the poster may belong (gender, race, etc.) | 1 | 2 | 3 | 4 | 5 | 6 | 7 | 8 | 9 |
| 4. The poster’s unique or distinctive characteristics | 1 | 2 | 3 | 4 | 5 | 6 | 7 | 8 | 9 |
| 5. Cues that could be used to locate the poster’s true identity | 1 | 2 | 3 | 4 | 5 | 6 | 7 | 8 | 9 |

Appendix C. Coding Criteria of Person-centeredness and Sample Messages

| Coding Criteria | Sample Messages |
| --- | --- |
| Level 1. Speaker explicitly criticizes, condemns, or challenges the target’s feelings or character.  Level 2. Speaker criticizes the target’s behaviors or supports the other party’s behaviors, feelings, or character.  Level 3. Speaker ignores the target’s feelings or asks the target to ignore his/her feelings. | “I don’t see why you think this is a tough decision. Don’t be childish! You are a grown-up know so make up your mind!” (Level 1) |
| Level 4. Speaker suggests or implies that the target may feel better about the situation or the situation may improve at some point in the future.  Level 5. Speaker expresses sympathy, understanding, or condolence without providing any legitimization or explanation of the target’s feelings.  Level 6. Speaker provides a non-feeling-centered explanation of the situation or attempts to help the target to reappraise the situation in order to reduce the target’s distressed emotional state. | “I completely understand where you are coming from. I had the same issue when deciding majors. I would suggest you ask yourself what your dream major is. Although it is hard, I am sure you will figure out!” (Level 5) |
| Level 7. Speaker provides an explicit legitimization of the target’s feelings.  Level 8. Speaker explicitly acknowledges the target’s feelings and provides an elaborated explanation of those feelings.  Level 9. Speaker helps the target to gain a perspective on his/her own feelings (feelings in the situation are explicitly recognized and legitimized/explained) and attempts to help the target to reappraise the situation, in order to reduce the target’s distressed emotional state. | “I understand where you are coming from. If I were you, I’d be very troubled too.; and want to quit right on the spot. However, instead of resulting with the final solution of quitting, I think you should try to save your internship first. First, try to confront your supervisor. Ask him why you were given only menial tasks, as if you haven't yet. Ask him why you were only given menial tasks, as well as why you were given no specific suggestions. Let him know you feel disappointed and offended, provided that it won't lead to greater problems. Also, let him know that you at least deserve an explanation. If you really value your internship, you should at least try to save it before quitting. I just hope that your supervisor does not ignore your confrontation, and works with you in solving the conflict that you two have. Sincerely, Edean91” (Level 9) |

Appendix D. Coding Criteria for Politeness and Examples

| Coding Criteria | Examples |
| --- | --- |
| 1. The participant uses informal address/greeting phrase at the beginning or during the response. | “Hey Marvelous45” |
| 2. The participant expresses agreement or approval of a message the support-seeker wrote in the posting. | “I know it can be frustrating to do such basic tasks when you want to learn, but it would probably be a good idea to stick it out anyway.” |
| 3. The participant uses in-group identity markers to convey in-group membership | “Some advice from a student intern herself: during your first week you will be asked to do things such file paper work, make copies, bring coffee, etc” |
| 4. The participant includes the support-seeker in the discussion by using 1^st^ person plural pronouns to refer to the writer or reader. | “Well, we all have to start from the bottom and work our way up” |
| 5. The participant uses discourse marker to show politeness. | “Please don’t stress” |
| 6. The participant uses joke (several sentences might be used to describe a same joke. In this case, you will count it as only one joke) or slang | “Your free labor is used for bitch work” |
| 7. The participant uses optimistic or encouraging expressions *unconditionally* | “Best of luck with finding a new one if this one doesn't work out!” |
| 8. The participant expresses sympathy or understanding of the support-seeker’s feelings or situation. | “I can totally relate to you” |
| 9. The participant uses explicit words or phrases to acknowledge the support-seeker’s competence or other positive attributes. | “It seems that your talents and/or skills are not being fully utilized, but do you have back-up options if you were to quit” |
| 10. participant tries to soften possible negative attributions about the support-seeker. | “Mistakes happen and it's disrespectful for him to treat you like” |
| 11. The participant gives reasons for the recommended behavior (e.g., explaining why the suggested action might work or be helpful *by explicitly using words or phrases to indicate causal relationship*) or asks for reasons for not doing something. | “Go above and beyond what you think is necessary, so that your employer will take you seriously” |
| 12. The participant asserts reciprocity | “Reply back if you have any more concerns” |
| 13. The participant uses formal address/greeting phrase | “Sincerely, Xxx” |
| 14. The participant is conventionally indirect by questioning the support-seeker’s ability or willingness to perform an act | “Would you want to do something that you have no passion for” |
| 15. The participant uses hedge to indicate that he/she is not assuming that the support-seeker will want to comply with what is recommended | “I suggest that you stick with the internship” |
| 16. The participant tries to minimize the imposition by using words to imply a lesser imposition on the hearer than it seems | “I say you give the internship a bit more time to see if it takes you anywhere” |
| 17. The participant explicitly shows deference by using words to abase him/herself or to raise the support-seeker’s status. | “My SAT scores were not as outstanding as yours.” |
| 18. The participant impersonalizes the situation or discussion by using general words to refer to the recommendation or advice | When interns are treated unfairly, they should keep working hard to gain others’ respects.” |
| 19. The participant explicitly apologizes or admits that he is impinging on the support seeker’s negative face (including thoughts and behaviors). | “I hate to say it, but you might be doing menial labor for quite some time before you get a "real" web design job, in this internship and in others” |
